# Prospective Role of Indigenous Leafy Vegetables as Functional Food Ingredients

**DOI:** 10.3390/molecules27227995

**Published:** 2022-11-18

**Authors:** Nyarai Mungofa, July Johannes Sibanyoni, Mpho Edward Mashau, Daniso Beswa

**Affiliations:** 1 Department of Life and Consumer Sciences, College of Agriculture and Environmental Sciences, University of South Africa, Science Campus, Johannesburg 1709, South Africa; 2 School of Hospitality and Tourism, University of Mpumalanga, Mbombela Campus, Mbombela 1200, South Africa; 3 Department of Food Science and Technology, Faculty of Science, Engineering and Agriculture, University of Venda, Thohoyandou 0950, South Africa; 4 Department of Biotechnology and Food Technology, Faculty of Science, University of Johannesburg, Doornfontein Campus, Johannesburg 1709, South Africa

**Keywords:** indigenous leafy vegetables, food security, processing practices, derivative foods, future of eating

## Abstract

Indigenous leafy vegetables (ILVs) play a pivotal role in sustaining the lives of many people of low socio-economic status who reside in rural areas of most developing countries. Such ILVs contribute to food security since they withstand harsher weather and soil conditions than their commercial counterparts and supply important nutrients such as dietary fibre, vitamins and minerals. Furthermore, ILVs contain bioactive components such as phenolic compounds, flavonoids, dietary fibre, carotene content and vitamin C that confer health benefits on consumers. Several studies have demonstrated that regular and adequate consumption of vegetables reduces risks of chronic conditions such as diabetes, cancer, metabolic disorders such as obesity in children and adults, as well as cardiovascular disease. However, consumption of ILVs is very low globally as they are associated with unbalanced and poor diets, with being food for the poor and with possibly containing toxic heavy metals. Therefore, this paper reviews the role of ILVs as food security crops, the biodiversity of ILVs, the effects of processing on the bioactivity of ILVs, consumer acceptability of food derived from ILVs, potential toxicity of some ILVs and the potential role ILVs play in the future of eating.

## 1. Introduction

Indigenous leafy vegetables grow naturally in open land, back yards and dumping sites [1]. They are plant species that are either genuinely native to a region or that were introduced to that region in the past and have since evolved through natural processes [2]. In rural settlements of developing countries, indigenous leafy vegetables (ILVs) are essential in boosting household food security and enhancing the quality of diets [3,4]. Several studies have reported that ILVs address gaps in nutrition by providing wholesome, reasonably priced and nutrient-dense food substitutes [2,3,5] due to their high concentration of essential nutrients [6,7]. Such nutrients include minerals (zinc, calcium, magnesium, iron and potassium), provitamin A, vitamin C and fibre [1,5,8,9]; in addition, ILVs are extremely low in fat and carbohydrates [1]. However, consumption of these vegetables is very limited as they have been marginalised in favour of exotic vegetables such as carrots, lettuce, tomatoes and others that are cultivated at an expense [2]. The few ILVs consumed include African kale, African eggplant, jute mallow, pumpkin leaves, slender leaf, African nightshade, cowpea, amaranth and spider plant [2,10]. In addition, ILVs can also be directly or indirectly used for medicinal purposes [3,11] due to their high phytochemical content [1,2].

Phytochemicals are known to possess antioxidant properties that are important in decreasing the likelihood of chronic and other non-communicable or age-related conditions, such as cancer, diabetes and cardiovascular disease [1,2,12]. Therefore, their high levels of essential nutrients, combined with their high phytochemical content, make ILVs suitable alternative functional food ingredients. In general, leafy vegetables are minimally processed (i.e., cut, shredded, etc.) and packaged before storage at low temperatures. Other processing methods include hot-air oven drying, freeze drying, sun drying, solar drying or hydro-thermal processing such as boiling, cooking and blanching [13,14,15]. In the case of ILVs, only sun and solar drying methods are currently practiced and, as a result, there are no ILV-derived products or ingredients available on the market. Thus, the use of locally available ILVs to develop new food products and ingredients could have a significant socio-economic impact on rural settlements since ILVs ingredients could easily be incorporated into drinks, soups and bakery products. Therefore, this paper aims to review the prospective role of ILVs as food ingredients.

## 2. The Role of Vegetables as Food Security Crops

Developing countries are faced with dire food security challenges. Despite having the largest amount of arable land available to feed its growing population, Africa continues to be the region with the worst food security [16]. It is predicted that the global population will be more than 9 billion by 2050 and an increasing world population presents a serious challenge to agriculture as it will contribute towards serious shortages of food, energy and water [17]. One of the most practical ways to tackle this challenge will be to increase knowledge of the many advantages surrounding the utilisation of ILVs [16].

Indigenous leafy vegetables are an important, readily accessible resource that may be used to attain food security and alleviate poverty in rural, semi-urban and urban areas [17,18,19,20]. This is because ILVs have a relatively higher nutritional value compared to exotic vegetables [19]. Even though ILVs have not been fully exploited in the quest to achieve food safety and alleviate malnutrition [10], many researchers agree that the vitamins, minerals, phytochemicals with strong antioxidant properties and nutritional (micronutrient) content in ILVs are their most valuable attributes [9,17,18,21]. The capacity of a food to provide nutrients is of great importance in food security [22]. 

Food security does not centre only on the availability and accessibility of food—it is also concerned about feedstocks that may also contribute an income to alleviate food insecurity as well as essential nutrients in correct proportions. Thus, lack of both economic diversity and nutritious foods plays a significant role in malnutrition and food insecurity in Africa where most rural and urban households depend on carbohydrate-rich diets which may increase the availability of energy but may not necessarily improve the nutritional status of consumers [23]. In contrast, such diets may be combined with micronutrient-rich foods such as ILVs that provide a rich source of micronutrients and health-promoting compounds that can supplement the nutritional value of these staple diets [24]. In addition, ILVs are very diverse, colourful and tasty foods that can be used to play a strategic role in achieving balanced diets [6]. Maize, potatoes, rice and wheat are among those crops high in carbohydrates that account for more than 60% of global dietary energy intake [25]. However, as human nutrition suffers because of overdependence and concentration on these food crops [23], dietary diversity should be encouraged as it qualitatively measures food consumption and indicates household food security status, particularly as this is a practical, cost-effective and sustainable means of alleviating food insecurity [4,17,25]. 

## 3. Biodiversity among Indigenous Vegetables

Indigenous leafy vegetables are diverse and include many different species [2,26,27]. Globally, over 7000 species of leafy vegetables are either cultivated or harvested from the wild for food [4,28]. Amongst these, ILVs that vary in shape, size, colour, taste and nutritional value are consumed in nearly all countries [9,25]. South Africa is home to over 100 species of wild and domesticated or cultivated ILVs including amaranth (*Amaranthus* spp.), cowpea (*Vigna unguiculata*), nightshade (*Solanum* spp.), spider plant (*Chlorophytum comosum*), lamb’s quarters (*Chenopodium album* Linn), purslane (*Portulaca oleracea* L.), blackjack (*Bidens pilosa*), jute mallow (*Corchorus olitorius*), Chinese cabbage (*Brassica rapa* L. ssp. *Pekinensis*), bitter melon (*Citrullus lanatus*), African cabbage (*Cleome gynandra*), pumpkin (*Cucurbita maxima*) [2,29,30,31,32,33,34,35,36,37,38,39] and slender leaf (*Crotalaria brevidens*).

The indigenous knowledge of a community determines the diversity of ILVs in a region and also influences the type of farming systems for ILVs, plant innovations that are practiced and the degree to which certain exotic plant species have been incorporated into the farming system [29]. Various factors, together with cultural disparities and the ancient inspirations of particular communities, have a substantial influence on the number of varieties of ILVs that are still in existence in different communities [10]. 

Numerous uses of ILVs have been documented, including those for food, cash crops, medicine, indigenous culture and ornamentation. Different types of ILVs exist, their definition as such being based on the utilisation of part of the plant, mostly leaves, shoot tips, fruit, seed, roots and flowers [19,40]. They can be prepared fresh or dried, depending on cultural preferences and the characteristics of the leaves or their immature seeds [41]. Indigenous leafy vegetables can be mixed together and eaten in various ways (e.g., as a relish, in soups and as part of a salad) [42]. Examples of ILVs consumed in South Africa and worldwide are presented in Table 1. 

## 4. Nutritional Composition and Health Benefits Associated with ILVs

Compared to exotic variants, ILVs offer a higher nutritional value [52]. Such vegetables would ensure an appropriate supply of those nutrients identified to be lacking if they are consumed in suitable proportions [53]. Since they contain significant amounts of vitamins, pro-vitamin A in particular [43], ILVs are highly beneficial since they maintain health and prevent diseases [53]. As an antioxidant, vitamin A plays an important role in preventing free oxygen radicals from causing damage to cells and, by so doing, reduces the incidence of some cancers, heart attacks, strokes, and maintains eyesight and the immune, skeletal, respiratory, reproductive, and integumentary (skin) systems [25,43]. However, due to the underutilisation of ILVs, there is little available information regarding their nutritional value [54].

Vitamin C is important for iron absorption in addition to maintaining the health of teeth and gums [9,25]. Folic acid decreases the likelihood of birth defects and vitamin K protects bones from osteoporosis and helps to contain inflammation [9,55]. Different studies have demonstrated that high folate intake from ILVs may decrease the risk of colon polyps by 30% to 40% [56,57,58]. Significant amounts of vitamin D, E, K, pantothenic acid, pyridoxine, niacin, folate, riboflavin and cyanocobalamin have been reported in ILVs [43]. Moreover, the carotenoids lutein, B-carotene, violaxanthin and neoxanthin are among those found in abundance in ILVs [59,60].

Table 2 shows the nutritional value of selected ILVs. Indigenous leafy vegetables are principal sources of dietary minerals including iron, zinc, calcium, magnesium, sodium, phosphorus and potassium [61,62,63]. When exposed to cooking and processing techniques, minerals are more stable than vitamins [64]. Minerals play a vital role in the metabolism of nutrients and inhibit degenerative diseases [53]. The potassium in ILVs assists in maintaining a blood pressure within a normal range [9]. In addition, ILVs serve as cheap alternative sources of protein [17], varying from 21 to 25 g/100 g. Thus, they play an important role in feeding the rural and urban low-income households because of their affordability compared to sources of animal protein (poultry, meat or fish) [65,66,67,68]. 

Additionally, ILVs are essential sources of health-providing antioxidants [6]. Compared to other food sources, ILVs contain more micronutrients and various amounts of compounds that are needed to address nutritional and health requirements [69]. Antioxidants in ILVs are a group of compounds that actively inhibit or delay the oxidation of lipids and other biomolecules, thus minimising oxidation damage to cells and that also assist in repairing cell damage [70]. They are, thus, an essential part of the defence system of the body [71]. Phenolic compounds including phenolic acid contribute to the antioxidant potential of many different plants [70,72,73]. Considering the bioactivity of these compounds and their presence in a variety of ILVs, dietary phenolics are viewed as natural antioxidants, with the vegetable sources providing them thus being considered to be functional foods [70]. 

**Table 2:** Nutritional content of selected raw indigenous leafy vegetables (mg/100 g).

ILV	Ca	P	Fe	Mg	Na	K	Vit C	References
Amaranth	323.70	89.00	7.50	122.00	230.00	341.00	50.00	[44]
Cowpea leaves	428.01	17.23	9.62	46.73	31.25	81.25	8.00	[66]
Nightshade	100.47	62.50	8.63	461.00	74.22	100.00	54.00	[44]
Slender leaf	1.234.40	11.25	28.13	155.00	22.66	162.50	-	[44]
Spider plant	1.484.40	48.95	29.67	47.50	18.75	75.00	-	[44]
Lamb’s quarters	309	72	1.2	34	43	452	80	[74]
Purslane	65	44	1.99	68	-	494	21	[74]
Blackjack	-	-	15	-	-	-	63	[75]
Jew’s mallow	208	83	4.76	64	-	559	37	[74]
Pumpkin leaves	15	41	0.87	15	4	170	43	[76,77]
Chinese cabbage	77	29	0.31	13	8	238	27	[78]

## 5. Functional Components of Indigenous Leafy Vegetables and Human Health

As indicated, ILVs are rich sources of polyphenols, flavonoids, amino acids, minerals, vitamins A and C, β-carotene and dietary fibre. These bioactive components are involved in protection against various conditions including cancer, diabetes mellitus, arthritis and cardiovascular disease [79]. Epidemiological studies have demonstrated that ILVs are effective in combating such health conditions and that this ability is related to their natural bioactive components [80,81,82] such as polyphenols and α-tocopherol being synthesised by plants to protect themselves against oxidative damage resulting from environmental stresses [83,84]. Table 3 shows the functional components of ILVs and their role in human health.

Polyphenols

Polyphenols are the most studied secondary metabolites in ILVs, being linked to various health benefits [85]. The composition of polyphenols may vary in different parts of the plants, so that phenolics such as catechins and quercetins may be generally present in vegetables while others may only be available in a particular species [86]. Moreover, the composition, content and biological characteristics related to polyphenols are influenced by plant phenology and changes in environmental conditions [87,88]. For example, ILVs such as amaranth and purslane have demonstrated changes in polyphenolic profiles and their related antioxidant activity at different stages of phenology [89,90]. Seven phenolic compounds including ferulic acid, p-coumaric acid, cinnamic acid, gentisic acid, caffeic acid, p-hydroxybenzoic acid and protocatechuic acid have been isolated in pumpkin leaves hydrolysed by subcritical water [91]. Of these, ferulic, p-coumaric, caffeic and gentisic acids are phenolics found at high concentration. Yields of phenolic acids, apart from gentisic acid, were high at a temperature of 160 °C, while high yields of gentisic acid required a temperature of 180 °C. However, the total phenolic content of hydrolysed pumpkin leaves was significantly reduced at temperatures above 160 °C and this was attributed to their decomposition at high temperatures. While hydrolysis temperatures above 160 °C decreased the levels of polyphenols, at the same time they increased antioxidant activity since higher temperatures result in the generation of antioxidant components from polyphenols [92]. 

The use of UPLC-QTOF/MS allowed the identification of 22 phenolic compounds in pumpkin leaves during blanching [93]. The major identified phenolic compounds were glycosylated (83.3%) and the remainder (16.7%) were tetracarboxylic acids and their derivatives. Simple phenolic glycosides accounted for 12.5% of the total phenolic compounds, but flavonoid O-glycosides had a higher value (41.7%) in terms of glycosylated phenolic compounds. Furthermore, 12.5%, 8.3% and 4.2% of the detected phenolic compounds were derivatives of glucuronic acid, hydroxycinnamic acid glycosides and isoflavonoid O-glycosides, respectively. The same authors indicated that raw pumpkin leaves had a higher value of total phenolic content (1.457.1 mg/kg) compared to the blanched samples. This shows that polyphenols from the leaf tissue are broken down during the blanching process [94].

**Table 3:** Functional components of indigenous leafy vegetables and their impact on human health.

Functional Components	Effect on Human Health	References
Phenolic acids: ferulic acid, p-coumaric acid, cinnamic acid, gentisic acid, caffeic acid, p-hydroxybenzoic acid and protocatechuic acid	These have anticancer and anti-inflammatory properties, provide protection against different diseases such as diabetes mellitus, osteoporosis, high blood pressure, arthritis, neurodegenerative disorders and headache, and influence the bioavailability of nitric oxide.	[95,96,97,98]
Flavonoids: myricetin, rutin, quercetin, kaempferol, delphinidin-3-O-glucoside, cyanidin-3-O-glucoside and quercetin 3-glucoside	These decrease oxidative stress to prevent hyperglycemia, act as an anti-inflammatory, prevent kidney failure, assist in cell growth control, are anticancer agents, have cardio- and neuro-protective properties, reduce the risk of neurodegenerative diseases, prevent stroke, have an antidiabetic effect and exhibit antiviral and antibacterial properties.	[99,100,101,102,103]
Carotenoids	These reduce the incidence of cataracts and cardiovascular disease, improve the immune response, reduce the likelihood of developing diseases such as cancer, muscular and degenerative diseases, contribute to the maintenance of cardiac cells, the kidney, and other organs and reduce the risk of type 2 diabetes and decrease metabolic syndrome.	[104,105,106,107,108]
Vitamin C	This inhibits cancer cell growth, reduces oxidation of low- and high-density lipoprotein, reduces oxidative stress and acts as an antihypertensive, boosts the immune system and is used to treat ailments such as scurvy and the simple cold and is involved in collagen synthesis.	[95,97,109,110,111,112]
Dietary fibre	This improves the digestion process, prevents cancer and is antidiabetic.	[113,114,115]

Mature amaranth and nightshade leaves contain proportionally higher amounts of total phenolics compared to young leaves, with values varying from 0.85 to 1.01 g/100 g for young compared to mature amaranth leaves, and from 1.09 to 1.29 g/100 g for young compared to mature nightshade leaves [116]. A similar increase in polyphenols according to leaf maturity was reported in three species of amaranth (*A. hypochondriacus*, *A. caudatus* and *A. cruentus*) [117]. Various plant parts were examined for their total phenolic content (TPC) and it was higher in the leaves compared to the seeds and stalks [118]. Moreover, an investigation of phenolics in *Amaranthus caudatus* during seven stages of growth identified seventeen phenolic compounds [90]. Rutin was the dominant compound in all stages of growth but there were changes observed with respect to its concentration during the growth cycle. Higher values were seen in the early and medium vegetative, early flowering and grain fill stages of plant growth, with the TPC ranging from 18.3 to 33.7 mg GAE/g extract. Lower TPC values were found in extracts from the shooting and budding stages. The authors suggested that elevated phenolic levels in the vegetative phases of amaranth may be associated with the predominant quantity of leaves to stalks that are available in these stages of plant growth in comparison to other stages. A two-year study (2018–2019) of polyphenolic compounds of nine amaranth species reported that the TPC of amaranth species varied from 8.38 to 116.0 mg GAE/g in 2018 and 100.1 to 141.9 mg GAE/g in 2019. The higher TPC levels noted in 2019 compared to those in 2018 indicated that climate variation contributed to the differences [119]. Sunlight is believed to increase phenolic levels, since photosynthesis influences the synthesis of polyphenols [120]. 

A study evaluated the phenolic compounds of purslane (*Portulaca oleracea* L.) in association with the stage of harvesting and parts of the plant and identified three phenolic acids (caffeic acid, sinapic acid hexoside and caffeic acid derivative) in extracts of purslane aerial parts [121]. Purslane leaves had higher levels of individual and phenolic compounds than the stems, irrespective of the stage of harvesting. Moreover, early harvesting at 29 days resulted in higher levels of phenolic compounds in the leaves. High levels of phenolic compounds during early stages of harvesting could be due to a protective role required during the growth of leaves since phenolic compounds are believed to represent the defence mechanisms of purslane. Similar results were reported where the composition of phenolic compounds in leaves was higher during the early stages of growth but decreased as the plant matured [122].

A study by Seong et al. [123] used HPLC to isolate and identify polyphenols in the outer, middle and inner leaves of Chinese cabbage (*Brassica rapa* L. ssp. Pekinensis). The TPC varied from 148.81 to 347.46 mg GAE/100 g with outer leaves having a higher level of TPC. The authors extracted hydroxycinnamic acid derivatives such as caffeic acid, ferulic acid, p-coumaric and sinapic acid from the Chinese cabbage leaves. Due to their ability to scavenge ROS and prevent lipid oxidation, the cinnamic acid and derivatives exhibit potent antioxidant properties [124,125]. All three types of leaves contained higher levels of sinapic acid since it is the principal phenolic acid in Chinese cabbage [126,127], ranging from 6.01 to 8.00 mg/100 g, while p-coumaric acid and myricetin ranged from 2.20 to 2.89 mg/100 g and 0.80–0.83 mg/100 g, respectively. However, caffeic and ferulic acids were observed in the outer Chinese cabbage leaves with values ranging from 1.39 to 0.47 mg/100 g. In another study, the phenolic acids of raw Chinese cabbage were quantitated using UPLC–Q-TOF/MS and quininic acid was found to have the highest concentration (209 mg kg^−1^) while myrectin-O-arabinoside showed the lowest concentration of 20.3 mg kg^−1^ [128].

The identity and concentration of phenolic compounds from seventeen genotypes of the spider plant (*Cleome gynandra* L.) from South Africa and beyond its borders were determined to show that the TPC varied from 9.86 to 12.21 mg GAE/g. There was no significant difference in terms of TPC according to spider plant genotypes from different agro-climatic zones in Limpopo and Mpumalanga Provinces, South Africa [129]. The concentration of polyphenolic compounds in spider plants is comparable to that of ILVs such as jute mallow, African nightshade and cowpea, most of which compounds contribute various health benefits [130]. The polyphenolic compounds of eight spider plant accessions were characterized during the vegetative, flowering and seed set stages. Higher TPC levels were obtained in the silique extract, followed by the flowers, leaves and stems at the flowering stage. Lower concentrations of phenolic compounds in parts of younger plants are thought to be associated with the low level of precursors of lignin in the cell wall because of low lignification of tissue in young plants [131].

Phenolic compounds were determined in the leaves of seven cowpea (*Vigna unguiculata*) cultivars planted in the Southern African region to show seven phenolic compounds as well as flavonoid glycosides including gentisic acid 5-O-glucoside, p-coumaric acid, four derivatives of quercetin, ferulic acid O-glucoside, and O-glucoside [132]. According to the results obtained from various sets of seventeen cowpea cultivars, the phenolic acid content of cowpeas varies significantly and depends on the phenotype, with values ranging from 34.6 to 376.6 mg/100 g of flour [133].

Flavonoids

Flavonoids are also secondary plant metabolites with a range of biological properties, including antioxidant, anti-microbial, anti-cancer, and anti-inflammatory effects [134]. Most plant tissue contains flavonoids [135] and flavonoid glycoside is abundant in leaves, flowers and fruits; aglycone is present in the woody tissue while flavonoid glycosides or aglycones are also available in the seeds [83]. According to the position of ring B, the level of oxidation as well as the cyclic condition of ring C, flavonoids can be divided into distinct categories including flavanols, flavonols, anthocyanidins, flavones, flavanones and isoflavones [136]. Myricetin, quercetin and kaempferol are the three main flavonoids found in the *Brassicaceae* family, which includes Chinese cabbage [137]. The total flavonoid content (TFC) of cowpea ranged from 0.95 to 0.36 mg quercetin equivalents/g, and the dark seed coat cultivars showed higher TFC levels than the white seed coat cultivars [138]. Major anthocyanins such as delphinidin-3-O-glucoside and cyanidin-3-O-glucoside were isolated and characterised in cowpea leaves. Various cowpea cultivars such as green, navy blue, black, grey and black/grey mottled have been reported to contain anthocyanins [139,140]. The lack of quantifiable anthocyanins in other coloured cowpea variants including red, maroon and brown demonstrates that the genetic component of cowpea’s anthocyanin production is important [141]. Nevertheless, the dominating anthocyanins in cowpea, regardless of genotype, are delphinidin-3-O-glucoside and cyanidin-3-O-glucoside [140,142]. Flavonoids were identified in the leaves of the spider plant, including quercetin, quercetin 3-glucoside and kaempferol 3-O-rutinoside [129]. The same authors indicated that two South African genotypes had higher quercetin levels than exotic genotypes. Moreover, South African genotypes were also superior with regard to quercetin-3-rutinoside.

Four flavonol glycosides were detected in *Amaranthus spinosus* L. of which two were identified as quercetin (Q)-3-O-rutinoside and Q-3-O-glucoside [143]. The third quercetin diglycoside that eluted before rutin appeared to contain hexose in addition to glucose and rhamnose or was a positional isomer of rutin. A kaempferol with a hexose and a deoxyhexose was identified as the fourth flavonol glycoside. It displayed a pseudomolecular ion of size m/z 593 and a notable fragment of m/z 285. The detection of flavonol glycosides was not reported in *A. spinosus* from a study that measured the TFC of 62 edible tropical plants [137]. Nine flavonoid constituents including six flavonols (rutin, kaempferol, quercetin, isoquercetin, hyperoside and myricetin) were detected in salt-tolerant amaranth vegetables. Of the six flavonols, quercetin and rutin were the most dominant followed by myricetin and isoquercetin [144]. For the first time, authors identified one flavanol (catechin), flavone (apigenin) and flavanone (naringenin) in salt-tolerant vegetable amaranth.

Vitamin C

The formation of collagen depends significantly on vitamin C (L-ascorbic acid) as well as the absorption of iron [145]. A study of leafy vegetables determined the influence of seasonal variation on the nutritional compositions of spider plants in southern Côte d’Ivoire to show that the vitamin C content of spider plants is higher in the rainy season (33.33 mg/100 g FW) than in the dry season (24.33 mg/100 g FW) [146]. The variation in vitamin C might be attributed to genetic factors, maturity level of the plant as well as the extent of exposure to the sun since high temperature destroys the vitamin [147]. The results of the seasonal study reflected higher vitamin C levels than those obtained in another study where the vitamin C content of spider plants was 13 mg/100 g FW [148]. However, both these study results indicate lower levels of vitamin C than those obtained in a study by Mibei and Ojije [149] in which the vitamin C content was 104.3 mg/100 g FW. A comparison of the vitamin C content of fresh, thermal- and nonthermal-processed leaves of Chinese cabbage and nightshade showed that the leaves of both plants were comparable in terms of vitamin C content whether they were fresh or freeze-dried. However, thermal and non-thermal treatments resulted in the loss of vitamin C with non-thermal (microwave) associated with a higher loss than conventional drying methods. This is thought to be linked to the utilisation of light energy during non-thermal treatment [14]. A comparison of the vitamin C content of young shoots or mature leaves of *Chenopodium album* L. showed a higher vitamin C content (5.6 mg/100 g) in the shoots compared to 5.2 mg/100 g in matured leaves [150].

Carotenoid content

The skin, skeleton tissues and respiratory organs all depend on carotenoid, which is a precursor of vitamin A [151]. The exposure of plants to environmental stress results in the biosynthesis of carotenoid [152]. Chinese cabbage has a total carotenoid content that ranges from 3.93 mg/100 g to 18.87 mg/100 g, which is in line with the patterns of other functional components. In *S. scabrum* leaves, the total carotenoid content varied from 586 to 691 µg/g on a DW basis, whereas in *S. retroflexum* leaves, the total carotenoid content was 0.733 µg/g on a FW basis [153,154,155]. The levels of β-carotene in the leaves of *S. nigrum* and *S. scabrum* ranged from 28.1 to 141.7 µg/g DW and from 55.1 to 96.0 µg/g DW, respectively. The highest amounts of total carotenoids were noted in *S. villosum*, which contained 138.11 µg/g DW, whereas *S. scabrum* contained 65.2 µg/g [155]. African nightshade has a higher amount of vitamin A (422 g retinol activity equivalent, RAE) than Jew’s mallow (329 g RAE) or pumpkin leaves [155,156]. Indigenous leafy vegetables supply more than 75% of the daily recommended amount for vitamin A.

Dietary fibre

The ILVs that have high fibre content include *Bidens pilosa*, which has 3770 mg/100 g, and *C. gynandra*, which has 1800–2100 mg/100 g [156,157]. Significant variations were noted in the dietary fibre content of twelve amaranths with the green morph genotype [158]. Genotype GRA9 had a higher value of 9.55 g 100/g FW, followed by GRA26 (8.56 g 100/g FW) and GRA4 (8.21 g 100/g FW). Genotype GRA11 showed the lowest value of 6.02 g 100/g FW, with an average value of 7.51 g 100/g FW. A study of the soluble and insoluble fibre content of spider plants during the dry and rainy seasons showed that soluble fibre was higher in the rainy season (16%) compared with 13.33% in the dry season. Moreover, insoluble dietary fibre content was higher in the dry season (25.1%) and lower in the rainy season (23.07%) [146]. The high levels of insoluble dietary fibre in spider plants is essential considering that dietary fibre enhances the metabolism of carbohydrate and lipid [159]. Moreover, the insoluble fibre of spider plants in the dry season meets the recommended fibre intake of 25 g/day for adults. Dietary fibre substantially contributes to alleviating constipation, slows down food digestibility and improves palatability. 

Other functional components of interest in ILVs include protein and amino acids. The protein content of amaranth species ranges from 13.57 to 19% [160,161,162]. Amaranth protein is rich in lysine and contains substantial amounts of iron, calcium, B vitamins, vitamin A, E and C [163]. Based on these nutritional properties, amaranth flour can be added to wheat flour to improve the nutritional value of baked products. Wheat flour has lower nutritional value than amaranth grain flour since its protein is deficient in essential amino acids such as lysine and threonine [161]. Therefore, amaranth flour has been utilised in baked wheat products such as bread, biscuits, muffins and other bakery products consumed in large amounts. However, these products have not yet been commercialised on a large scale.

Like other legumes, cowpea leaves are a rich source of proteins, containing 23–40% protein on a dry basis; they are storage proteins, and their main purpose is to provide nutrients to the germination of the seed [164,165]. The storage proteins of pulses, including cowpea, contain amino acid sequences that, when digested, release peptides that may have additional bioactive properties, such as acting as angiotensin I-converting enzyme (ACE) inhibitors and antioxidants, among others [166,167]. Peptides are typically made of three to twenty amino acid residues that are released due to the enzymatic proteolysis of different animal and plant proteins [167]. Peptides that are referred to as functional or biologically active compounds have been reported to have antimicrobial, anti-hypertensive, antioxidative, anti-dyslipidemic, anti-carcinogenic and anti-diabetic properties [168,169,170,171,172]

Compared to amino acids containing sulphur, cowpea proteins have higher amounts of valine, leucine, phenylalanine and lysine and these vary according to the genotype [173,174]. Gupta et al. [174] evaluated the amino acid content of seven genotypes of cowpea, where the minimum and maximum total essential amino acid content was 27.50 and 33.43 g of protein. Cowpea flour has been utilised to improve the nutritional properties of various food products. For example, Ritika et al. [175] incorporated malted and fermented cowpea flours (up to 20%) in wheat flour noodles. The incorporation of cowpea flour improved the protein content as well as reducing the cooking time and hardness of the noodles.

## 6. Selected Analytical Methods for Determining the Phytochemicals of Indigenous Leafy Vegetables

Various analytical methods are used to determine the bioactive compounds and antioxidant activity of ILVs. However, the amount of antioxidant in ILVs differs in terms of the extent of their activity and mode of action and this contributes to the difficulty in their analysis [82]. Although there are different methods of measuring antioxidant activity, each has drawbacks and makes it difficult to compare results because of the variety of reaction mechanisms and phase locations [176]. Moreover, the main factors, such as temperature and solvent, that affect the extraction conditions must be optimised to obtain extracts with the highest extraction yield, antioxidant capacity and bioactive compounds. Therefore, sample preparation is an important step in the analysis of antioxidants and bioactive compounds in ILVs [177]. Standard methods for the analysis and determination of antioxidant capacity in ILVs include Trolox equivalents of antioxidant capacity (TEAC), 2′-Azinobis (3-ethylbenzothiazoline 6-sulfonate) (ABTS) antioxidant activity, DPPH radical scavenging assay, Ferric-reducing antioxidant power and vitamin C equivalent antioxidant capacity. The principles of these methods differ, for example, TEAC measures the ability of antioxidants to scavenge the stable radical cation ABTS^+^ (2,2′-azinobis (3-ethylbenzothiazoline-6-sulfonic acid)), a blue-green chromophore with maximum absorption at 734 nm that decreases in its intensity in the presence of antioxidants. The ABTS assay is based on the decolourisation that occurs when the radical cation ABTS^+^ is reduced to ABTS’ (2, 2′azino-bis (3-ethylbenzthiazoline-6- sulphonic acid). 

The Folin–Ciocalteu is a standard method used to determine the total phenolic and flavonoid contents of ILVs. Methanol is commonly used during the extraction of the ILV samples. In order to identify extracts of phenolic compounds of ILVs, high-performance liquid chromatography mass spectrometry (HPLC-MS/MS) analysis was carried out using a liquid chromatography (LC) system coupled with a quadrupole ion trap mass spectrometer. The β-carotene was also identified using the HPLC system software by comparing the retention time (RT) of an unknown peak with the reference standard. In addition, recent mass spectrometer equipment such as liquid chromatography-tandem mass spectrometry (UHPLC-ESI-q-TOF-MS/MS) and an ultra-high-pressure liquid chromatography (UHPLC) system equipped with quadrupole time-of-flight (QTOF) mass spectrometer (MS) were also employed to identify and quantify the predominant polyphenolic acids of ILVs [14,178].

The development of environmentally friendly methods for extracting high-value chemicals and extracts with biological activity from natural sources such as ILVs has drawn more attention in recent years. In line with this, green extraction technologies such as pressurised liquid extraction (PLE) and hot water extraction (PHWE) have been adopted as efficient techniques for extracting phytochemicals from plant tissues such as ILVs, depending on the extraction solvent employed [179,180]. The use of GRAS solvents (generally recognized as safe) in green extraction methods like those based on PLE and PHWE ensures the absence of harmful solvents in the final ingredients and products obtained [178]. Even though PLE can be carried out using a variety of solvents, ethanol and water are preferred for green extractions. On the other hand, PHWE utilises water as the extraction solvent, which is readily available and compatible with most chromatographic instruments [181].

## 7. Influence of Processing Methods on the Nutritional and Functional Components of Indigenous Leafy Vegetables

Indigenous leafy vegetables are extremely perishable; they begin to lose their freshness as soon as they are harvested and keep doing so until they are eaten [13]. When handled poorly, ILVs are quickly damaged and types such as amaranth are very susceptible to wilting. The loss of moisture during handling and storage is one of the post-harvest issues that results in weight loss [25]. Extending the shelf life of the ILVs through processing is a crucial opportunity to prevent needless losses [13]. Because ILVs are mostly produced during the rainy season and their production is seasonal, storing them will guarantee that these vegetables are accessible all year round and should maintain a reasonable level of quality [10]. 

Any modifications to raw materials used to make food, including meal preparation, are referred to as food processing. Processing is done to make food palatable, lower the amount of plant natural toxins and to prevent losses due to spoilage from autolysis or microbial attack [15]. Thus, food processing is a crucial stage in the production process that can be utilised to ensure food safety, maintain quality, increase shelf life and avoid spoilage [182]. Traditional food processing methods serve the objective of preservation since they ensure a year-round supply of health-providing, nutrient-dense food, especially during times of scarcity [183]. Additionally, it provides advantages in terms of enhancing the handling of produce, decreasing food losses and improving the value of the final product [15]. Processed ILVs offer health and nutritional benefits akin to those of fresh vegetables; they retain a comparatively decent taste compared to fresh ILVs, are simple to prepare, widely accessible, easy to handle and expand when cooking. Because of this, they can be utilised to feed a large household [13].

There are several ways of processing food, including vacuum sealing, drying, canning and fermentation [13]. The indigenous methods in use, such as drying and fermentation, are time-tested and have been used over generations to preserve produce after harvest [16]. Table 4 shows the influence of different processing methods on the functional components and nutritional composition of ILVs. Vegetables that have been cut into small pieces and blanched may then be preserved via dehydration or sun drying on open surfaces in direct sunlight [10]. However, direct sunlight has been shown to deteriorate food colour, vitamin content and flavour, and there is a chance that contact with dust, dirt and insect pests could contaminate such food [15]. 

Fermentation is known to be very effective in eliminating anti-nutritional factors such as phytic acid and glucosinolates [93]. It is regarded as a practical and affordable domestic food preservation technique used to prepare nutrient-rich food with improved palatability, taste, aroma, texture and storage quality, and overall to enhance the nutritional content and the safety of food products [10]. Food processing, on the other hand, can turn vegetables from perishable produce into staple foods with an extended shelf life, facilitating their global transit, distribution and availability [10]. 

## 8. Incorporation of Indigenous Leafy Vegetables as Functional Ingredients in Selected Food Products

Despite being consumed as food, ILVs have generally been perceived as weeds [3], low-value vegetables [32] and are restricted to certain areas or communities. Their lower level of utilisation can be associated with the perception that ILVs are food for hard times and for low-economic-status households. As a result, the advantages, and economic benefits of ILVs may not be fully realised unless such perceptions surrounding these vegetables change [191]. Several research efforts of incorporating ILVs into popular food formulations are presented in Table 5. Research findings on consumer acceptability and the nutritional quality of food products that had been enhanced with ILVs is also highlighted in Table 5.

## 9. Potential Toxicity of Some Traditional Leafy Vegetables

The presence of phytochemical secondary metabolites makes edible plants potentially toxic [200]. These compounds are not necessary for the survival of the plant, but they are produced to improve the plant’s ability to fend off predators or interact with its surroundings, herbivores and/or symbiotic insects [201]. For example, *Solanum* species are among ILVs that contain toxic alkaloids such as glycosides of solasodine and solanidine [154]. In addition to the presence of secondary metabolites, ILVs tend to acquire higher amounts of heavy metals which are known to be micronutrients in minute quantities [202]. 

Some of the micronutrients that are vital for the growth and development of the plant as well as productivity may become toxic when their content levels exceed permissible limits [203]. Such micronutrients include heavy metals such as copper, iron, Molybdenum and zinc [204]. Consuming contaminated ILVs can expose consumers to heavy metals which are harmful and pose a severe health risk because they can cause malnutrition, immune system fragility, mental growth retardation and gastrointestinal cancer [205,206]. High levels of Cu and Zn may cause oxidative stress through redox reactions [207]. Therefore, there is a need to measure their concentration in plants as their deficiency and presence in unacceptable levels cause a health risk to humans.

## 10. Indigenous Leafy Vegetables’ Potential Role in the Future of Eating

Edible indigenous plant species have formed a significant part of the human diet for centuries. However, there is a concern about the decline in their utilisation in food applications [208]. This decline can be attributed to the introduction of exotic vegetables [3]. The majority of people today are not aware of the benefits of ILVs, despite the fact that historically they have been a component of indigenous societies and traditional diets. Thus, ILVs are in danger of being lost in Africa as they are being replaced. There are insufficient seeds and a lack of information about their performance and input requirements [209]. Even though cultivation of ILVs has decreased and is still declining globally, these crops provide increased genetic diversity and have the prospect to increase food and nutritional security [210]. 

According to the concept of meal cultures, cultural, socio-economic and gender factors influence food intake. However, utilising these factors to analyse ILV consumption demonstrates that it is a complicated issue that is influenced by the availability of resources, expertise of food preparation and cooking, gender relations, and beliefs [211]. Unfortunately, knowledge on ILVs is rapidly being lost, especially among younger people who have the impression that ILVs are primitive, old-fashioned and that farming is an unpopular vocation [212]. The use and harvesting of ILVs have declined due to lack of information regarding their cultivation, nutritional composition, cooking and preservation methods, and, without cultivation, these factors might potentially result in genetic erosion and possible loss of biodiversity of food plants such as ILVs [3]. Nowadays, these plants are less competitive since commercial farming, research and development have mostly ignored them [4]. Sadly, these vegetables are disappearing from local diets around the world, and this has a negative impact on their potential role as valuable food ingredients [213]. 

Due to decades of neglect by academics and farmers in favour of staple crops, ILVs have had to endure over time, frequently in challenging conditions, without much assistance from humans. As a result, ILVs have evolved to resist unfavourable environmental factors including drought stress [214,215]. Therefore, ILVs can contribute to guaranteeing future food security because they have developed to be drought-tolerant [216].

The consumption of ILVs in countries such as South Africa is location-based and varies greatly; there is awareness among many people who consume them because they believe these vegetables will reduce the risk of certain diseases [4]. Hence, consumer health consciousness should help stimulate a move in the direction of ILV utilisation. Indigenous leafy vegetables are now acknowledged as future crops due to their numerous advantages, including their medicinal properties, therapeutic uses and climate resilience [17]. Indigenous leafy vegetables are nutritionally dense, economically viable and locally available, meaning that they qualify as future smart foods [21]. Increasing the amount of ILVs in the diet will be beneficial to achieve zero hunger. The relatively untapped potential of ILVs could very well hold the answer to future eating and nutrition security [210]. In the years to come, it will be interesting to note ILVs involvement in the official food system, especially to see if it results in a change in urban food culture [217]. Lastly, there is a need to address the fast-dwindling knowledge of how to acquire, grow, prepare, conserve and store these vegetables.

The recent global economic crisis, steep food and fuel prices and interest rate increases have put pressure on regular consumers who are already having difficulty meeting their basic demands, causing severe food insecurity [218]. Poverty, lower levels of maternal education, larger household sizes and unemployment have also increased the risk of food insecurity [219]. According to research, an adult’s food insecurity is associated with negative health outcomes like obesity, chronic disease and mental illness. Additionally, there is a connection between food insecurity, slowed growth, inadequate general development and diminished academic performance. Another aspect contributing to nutritional problems is the rising trend of people consuming more inexpensive, fast foods and less healthy foods [219].

There are conflicting study reports, but they all support the idea that eating these traditional vegetables may provide medical benefits [10]. According to studies, ILVs contains carotenoids and a diet rich in carotenoids helps prevent the onset of some cancers, including lung, skin, uterine, cervical and gastrointestinal cancers, as well as macular degeneration, cataracts and other conditions linked to oxidation and free radical damage [220]. In addition, ILVs have the potential to promote healthy immune systems in reducing the risks associated with blood pressure and cardiovascular diseases [221,222]. Indigenous leafy vegetables are not only important for nutrition and may be used as condiments in traditional cuisine [222], but they also have the potential to improve traditional medicine, provide an income for rural populations and create employment [17,223]. 

## 11. Conclusions

Indigenous leafy vegetables have been part of the human diet and recognised for their valuable attributes by contributing to household food security and consumer health, generating dietary diversity, creating employment opportunities and alleviating malnutrition and poverty. However, indigenous knowledge regarding their importance is contested and increasingly becoming scarce and inadequate due to the small quantities of these vegetables being used to augment diets. As such they do not play a significant role in food security and consequently this may enhance their disappearance as a nutritional food source. Lack of seeds has also resulted in low yields and some types of ILVs becoming extinct, which adds to a decreased consumption of ILVs. As ILVs are still neglected, unrecognised, unappreciated, undervalued, viewed as shameful to eat, as a poor man’s meal, and being out-of-date, several measures should be put in place by all sectors to ensure their continued availability. There is an urgent need to educate the population on the inherent potential of indigenous vegetables in order to make them an important part of mainstream diets which would enhance food security, prevent malnutrition and generate income. Generally, ILVs contain high amounts of nutrients such as minerals and provide an inexpensive and valuable source of nutrition. Diversification of these crops is essential if the world is to have secure food supplies. In addition, ILVs are extremely perishable, and their quality begins to deteriorate as soon as they are harvested and continues through processing until they are consumed. Therefore, post-harvest processing methods need to be developed and promoted to improve handling, minimise post-harvest losses, increase shelf-life and add value to these vegetables. Furthermore, cultivation and consumption of ILVs should be promoted and indigenous knowledge on various preparation, food processing and cooking methods should be disseminated, in order to enhance their utilisation. It is recommended that authorities promote these ILVs for household food security, dietary diversity, creating employment opportunities, malnutrition and poverty alleviation.

## Figures and Tables

**Table 1:** Selected indigenous leafy vegetables and their respective edible parts consumed in South Africa and globally.

ILV	Scientific Name	Local Name	Family	Parts Consumed	References
Lamb’s quarters	*Chenopodium album*	*Imbilicane*	Chenopodiaceae	Leaves and young shoots	[43]
Amaranth	*Amaranthus hybrids*	*Unomdlomboyi*	Amaranthaceae	Leaves	[44]
Purslane	*Portulaca oleracea*	*Igwanitsha*	Portulaeaceae	Succulent stems and leaves	[43]
Blackjack	*Bidens pilosa*	*Mothagaraga*	Asteraceae	Young tender shoots and leaves	[45,46,47]
Nightshade	*Solanum retroflexum*	*Umsobo*	Solanaceae	Leaves and tender shoots	[44]
Jew’s mallow	*Corchorus olitorius*	*Delele*	Tiliaceae	Leaves and fruit	[48,49]
Pumpkin	*Cucurbita maxima*	*Mpodi*	Cucurbitaceae	Leaves, fruit, young shoots, flowers and seeds	[45]
Chinese cabbage	*Brassica rapa*	*Isiqwashumbe*	Brassicaceae	Leaves	[45]
Cat’s whiskers	*Cleome gynandra*	*Amazonde*	Capparaceae	Leaves and tips	[50]
Cowpea	*Vigna unguiculata*	*Dinawa*	Fabaceae	Young shoots and leaves	[51]

**Table 4:** Influence of processing methods on the functional components of indigenous leafy vegetables.

Indigenous Leafy Vegetable	Processing Method	Effect on the Functional Components and Nutritional Value	Reference
Nightshade	Solar drying	Increased the leaves’ overall carotenoid content by 40%	[14]
		Increased phenolic acids such as caffeoylmalic acid, rutin and kaempferol-3-O-rutinoside in the leaves	[14]
		Increased the chlorogenic and neochlorogenic acid levels in leaves	[14]
		Improved FRAP activity	[14]
	Fermentation	Improved amounts of vitamins B1, B2 and C of the leaves	[184]
		Increased TPC and phenolic compound bioavailability, especially for phenolic acids and flavonoids in the leaves	[185]
	Steaming or cooking in plain hot water	Enhanced the levels of hydroxycinnamic acid derivatives and caffeoylmalic acid	[14]
	Steam blanching (water or lemon juice)	Increased the level of caffeoylmalic acid	[14]
	Stir frying	Increased the amounts of 3-caffeoylquinic, 4-caffeoylquinic and 5-caffeoylquinic acids, Ekaempferol-3-O-rutinoside, chlorogenic acid, caffeoylmalic acid and quercetin-3-O-xylosyl-rutinoside	[14]
Nightshade pickle and relish	Fermentation	Improved the β-carotene content of both products	[186]
Jew’s mallow	Cooking	Improved mineral content (phosphorous, potassium, calcium, magnesium, sodium and micro minerals (iron, manganese, zinc)) of the leaves	[187]
		Enhanced protein, ash and dietary (soluble and insoluble) fibre	[187]
		Improved cellulose and hemicellulose of the leaves and enhanced essential amino acids except phenylalanine	[187]
Chinese cabbage	Blanching in 5% lemon juice	Increased total chlorophyll retention	[128]
		Increased TPC, FRAP and TEAC	[128]
		Improved quinic and ferulic acids	[128]
		Improved levels of kaempferol-dihexoside, sinapoyl malate, rutin, isorhamnetin-O-dihexoside and kaempferol-3-O-hydroxyferuloyl-trihexoside in the leaves	[128]
	Stir frying	Improved levels of kaempferol-3-O-hydroxyferuloyl-trihexoside, kaempferol-dihexoside, sinapoyl malate, rutin and isorhamnetin-O-dihexoside	[128]
	Fermentation	Increased β-carotene content	[188]
		Improved mineral content (iron, zinc, calcium, potassium, copper and nickel)	[188]
Pumpkin leaves	Boiling	Improved the bioaccessibility of most polyphenols apart from methylquinic acid, cis-4-feruloylquinic acid and phenethyl rutinoside	[189]
	Stir frying	Enhanced the release and bioaccessibility of β-carotene as well as antioxidant activities	[189]
		Significantly improved the FRAP activity	
	Steam blanching (plain water)	Retained TPC and minimised the loss of quercetin	[93]
		3-glucoside 7-rhamnoside, kaempferol 7-neohesperidoside, isoorientin 2′′-O-rhamnoside, isorhamnetin-3-O-rutinoside, quercetin 3-galactoside, coumaroyl glucaric acid, isorhamnetin-3-galactoside-6′′-rhamnoside, 2-caffeoylisocitric acid and quercetin 3-galactoside 7-rhamnoside	
		Enhanced antioxidant capacity (FRAP and ABTS).	
Amaranthus leaves	Fermentation	Improved the mineral content (calcium, magnesium, zinc, iron, selenium and copper)	[190]

TPC = total phenolic content.

**Table 5:** Consumer acceptability and nutritional quality of food products enhanced with indigenous leafy vegetables.

Indigenous Leafy Vegetable	Food Products	Effects on Consumer Acceptability and Nutritional Quality	References
*Amaranthus*	Provitamin A-biofortified maize extruded snacks	Consumer acceptability was negatively affected at higher amaranth concentrations (3% *w*/*w*) but incorporation of amaranth leaf powder improved the essential amino acid, provitamin A and iron content of extrudates.	[192]
*Vigna unguiculata* (cowpea) leaves	Porridge	Porridge enhanced with cowpea leaf powder was less acceptable than plain cereal porridge even though they were rated equally high by consumers in terms of their willingness to purchase.	[193]
*Solanum nigrum* (nightshade) leaves	Soup	The formulation with 4% leaf powder, 30% starch and 66% spice mix was more accepted by consumers with regard to colour, flavour, taste and mouth feel. The soup contained higher levels of polyphenols, antioxidant content, crude protein, carbohydrates and crude fibre.	[194]
*Chenopodium album* Linn (lamb’s quarters)	Green gram *dal* and *paratha* (conventional foods)	Those foods that contained 7% or 5% dehydrated *Chenopodium album* leaves were most accepted by consumers. The final products had higher iron content than the control sample.	[195]
*Portulaca oleracea* (purslane)	Bread	Bread samples with low concentration of purslane powder were highly acceptable compared to the bread sample with 15% purslane powder.The proximate composition of purslane-enriched breads was significantly improved compared to the control.	[196]
*Bidens pilosa* (blackjack)	Raw ground beef	Used to reduce lipid oxidation during storage period.	[197]
*Cucurbita maxima* (pumpkin) leaves	Cassava pasta	The consumer appeal of cassava pasta with pumpkin leaf powder was negatively affected as it was less acceptable compared to the yellow pasta (control).Substantially higher content of protein, fibre, ash, beta-carotene, iron and zinc was reported.	[198]
*Brassica rapa* L. ssp. *Pekinensis* (Chinese cabbage) outer leaf powder	Muffin	Increased hardness of the muffins resulted in lower acceptance score for texture. Flavour was also affected by high leaf powder levels as shown by lower acceptance score compared to the control. Overall, the sensory attributes of muffins with up to 2% of Chinese cabbage dietary fibre were acceptable. A substantial increase in dietary fibre content and high antioxidant activity of the muffins compare to the control were observed.	[199]

## Data Availability

There are no data outside that reported in this article.
